# Diagnostic and prognostic significance of cell death markers in patients with cirrhosis and acute decompensation

**DOI:** 10.1371/journal.pone.0263989

**Published:** 2022-02-17

**Authors:** Philipp Stoffers, Sabrina Guckenbiehl, Martin Walter Welker, Stefan Zeuzem, Christian Markus Lange, Jonel Trebicka, Eva Herrmann, Christoph Welsch

**Affiliations:** 1 Department of Internal Medicine 1, Goethe University Hospital Frankfurt, Frankfurt am Main, Germany; 2 Department of Gastroenterology and Hepatology, University Hospital Essen and University of Duisburg-Essen, Essen, Germany; 3 Institute of Biostatistics and Mathematical Modelling, Faculty of Medicine, Goethe-University, Frankfurt am Main, Germany; University of Navarra School of Medicine and Center for Applied Medical Research (CIMA), SPAIN

## Abstract

**Background:**

The transition from compensated to decompensated liver cirrhosis is a hallmark of disease progression, however, reliable predictors to assess the risk of decompensation in individual patients from routine diagnostics are lacking. Here, we characterize serum levels of cell death-associated markers and routine biochemistry from patients with chronic liver disease with and without decompensation.

**Methods:**

A post-hoc analysis was based on prospectively collected clinical data from 160 patients with chronic liver disease, stably compensated or decompensated at baseline or during follow-up, over a median period of 721 days. Serum levels of damage-associated molecular patterns (DAMPs) and routine biochemistry are quantified at baseline (for all patients) and during follow-up (for patients with acute decompensation). The panel of DAMPs assessed in this study comprises high-mobility group-box protein 1 (HMGB1), cytochrome C (cyt C), soluble Fas-ligand (sFasL), interleukin 6 (IL-6), soluble cytokeratin-18 (CK18-M65) and its caspase‐cleaved fragment CK18-M30.

**Results:**

In this cohort study, 80 patients (50%) were diagnosed with alcoholic liver cirrhosis, 60 patients (37.5%) with hepatitis C virus- and 20 patients (13.5%) with hepatitis B virus-related liver cirrhosis. At baseline, 17 patients (10.6%) showed decompensated liver disease and another 28 patients (17.5%) developed acute decompensation during follow-up (within 24 months). One hundred fifteen patients showed stable liver disease (71.9%). We found DAMPs significantly elevated in patients with decompensated liver disease *versus* compensated liver disease. Patients with acute decompensation during follow-up showed higher baseline levels of IL-6, sFasL, CK18-M65 and–M30 (*P*<0.01) compared to patients with stably compensated liver disease. In multivariate analyses, we found an independent association of baseline serum levels of sFasL (*P* = 0.02; OR = 2.67) and gamma-glutamyl transferase (GGT) (*P*<0.001; OR = 2.1) with acute decompensation. Accuracy of the marker combination for predicting acute decompensation was high (AUC = 0.79). Elevated aminotransferase levels did not correlate with decompensated liver disease and acute decompensation.

**Conclusions:**

DAMPs are elevated in patients with decompensated liver disease and patients developing acute decompensation. The prognostic value of a marker combination with soluble Fas-ligand and GGT in patients with liver cirrhosis should be further evaluated.

## Introduction

Accelerated cell death in chronic liver disease can lead to liver cirrhosis and its complications [1, 2] The most common causes underlying liver cirrhosis in Europe are alcohol abuse and chronic viral infections, i.e. hepatitis b and c virus infections [3, 4]. Worldwide liver cirrhosis is diagnosed in more than 600.000 patients each year totaling in about 300 million patients [5]. Liver cirrhosis is associated with high mortality and rank 14^th^ among the most frequent causes of deaths around the world [6]. The natural history of liver cirrhosis is characterized by an asymptomatic (compensated) phase followed by a (rapidly) progressive phase marked by the development of complications [2]. The most frequent overt complications of liver cirrhosis are ascites, upper gastrointestinal bleeding, encephalopathy, and jaundice [7]. Patients with decompensated liver disease show median overall survival of only two years and an almost 4-fold increased risk of death during the following year [8, 9]. Early diagnosis and close monitoring for disease progression and decompensation are vital in the management of patients with end-stage liver disease.

In clinical routine, alanine aminotransferase (ALT) levels are used to assess necro-inflammation and estimate the risk of disease progression. However, ALT is not specific for liver cell damage [10, 11] and there is evidence that elevated ALT levels not necessarily correlate with the degree of histological liver injury [12, 13]. The capability of specific markers of inflammation to improve CLIF-C AD prediction of mortality in acute decompensation of the liver was shown in the original CANONIC cohort [14]. However, blood-based parameters to assess disease progression and potentially identify patients at risk for hepatic decompensation are lacking. Cell-death responses are important drivers of liver disease progression [15–17]. Damage-associated molecular patterns (DAMPs) are released to the extracellular space and possess pro-inflammatory potential [16, 18, 19]. Several DAMPs are previously characterized in chronic liver disease. The high-mobility-group box protein 1 (HMGB1) is passively released by dying hepatocytes, causing inflammation and activation of macrophages [20, 21]. Intracellular stress can cause cytochrome C from the inner layer of the mitochondrial membrane in hepatocytes to translocate to the cytosol and extracellular space, causing apoptosis in neighboring cells [20–24]. The huge amount of Fas receptor expressed on the surfaces of hepatocytes suggest a prominent role of Fas-induced apoptotic cell-death in liver disease. Levels of soluble Fas-ligand (sFasL) are reported to correlate with liver damage [25]. Moreover, previous studies showed increased serum levels of interleukin 6 (IL-6) related to liver disease progression and found correlations of IL-6 levels with mortality [26–29]. Although IL-6 is a cytokine it is also considered a DAMP due to its ability to induce inflammation and cell death when released from necrotic cells [30]. During apoptotic cell death, cytokeratin-18 from hepatocytes is cleaved by caspases and released from the dying cells, CK18-M65 and–M30 fragments [17, 31].

Given the close relation between cell-death markers and liver disease progression, we hypothesized that levels of circulating DAMPs might be of prognostic value in advanced-stage liver disease to identify patients at risk for the development of acute decompensation. We assessed serum levels of DAMPs in a cohort of patients with chronic liver disease and examined differences in the serum levels of DAMPs between patients with compensated and decompensated disease and acute decompensation.

## Materials and methods

### Patients

From 2017, patients presenting with liver cirrhosis at our outpatient department at Goethe-University Hospital Frankfurt, Germany, were consecutively enrolled into a cohort study. Inclusion criteria were age ≥ 18 years, liver cirrhosis (different etiologies), and written informed consent to participate in the study. Exclusion criteria were age < 18 years, pregnancy or breastfeeding, hepatocellular carcinoma (HCC), infection with human immunodeficiency virus (HIV) or therapy with immunosuppressive agents. The patients were followed every three months (routine surveillance of patients with liver cirrhosis in our outpatient clinic). At each follow-up time point, clinical characteristics and routine laboratory data were recorded and serum samples were stored for further analyses. Liver cirrhosis was assessed by shear-wave elastography (Siemens Acuson S2000TM system; pSWE (ARFI) Virtual Touch Quantification (VTQ); F4 ≥ 1.8 m/s) and corresponding laboratory and/or radiological findings (e.g. ultrasound showing splenomegaly). Acute decompensation of liver cirrhosis was diagnosed according to the acute-on-chronic liver failure (ACLF)-criteria proposed by the CLIF-EASL consortium (i.e. clinical findings of ascites, hepatic encephalopathy or gastro-intestinal bleeding) [32].

### Ethics approval

The study is approved by the local ethics committee of the Goethe-University Hospital Frankfurt. For the retrospective analysis, all data were anonymized and deidentified. No informed consent was required for the retrospective analysis (HIC approval no. 314/13).

### Blood sampling

Blood was taken from each individual at the day of inclusion into the study (baseline) and during follow-up visits at 3-month intervals (see S5 Fig). Serum levels of DAMPs, that is high-mobility group-box protein 1 (HMGB1), cytochrome C (cyt C), soluble Fas-ligand (sFasL), interleukin 6 (IL-6), soluble cytokeratin-18 (CK18-M65) and its caspase‐cleaved fragment CK18-M30, and routine biochemistry and hematology were assessed at baseline and at the time of AD. Serum tubes were centrifuged at 3000 rpm for 10 min and the supernatants were collected. Serum supernatants and EDTA samples were aliquoted and stored within 4 hours of collection at -80°C until further use. MELD-Na was calculated as part of the routine blood sampling: MELD + 1.32 x (137-Na)–[0.033 x MELD x (137-Na)] [33].

### Quantification of DAMPs

Caspase-cleaved CK18 was measured in serum samples using the M30 Apoptosense and M65 ELISA two-side enzyme-linked immunosorbent assays (PEVIVA AB, Bromma, Sweden). IL-6, HMGB1, sFasL and cyt C levels were measured with commercial enzyme-linked immunosorbent assay kits (Lifespan Biosciences, Washington, USA) according to the manufacturer’s instructions. Undiluted serum samples showed sFasL and IL-6 levels out of the upper range of the ELISA. Therefore, serum samples were diluted with RNAse-free water by 2-fold and 5-fold respectively for sFasL and IL-6 quantification. Absorbance was measured at 450 nm on an EnVision 2104 Multilabel plate reader (Perkin Elmer).

### Statistical analysis

Statistical analyses were conducted using BiAS (Version 11.09, Epsilon-Verlag, Darmstadt, Germany) and GraphPad Prism (Version 8, GraphPad Software Inc, California). Group differences were assessed by Wilcoxon-Mann-Whitney-U test and Kruskal-Wallis test. *P-*values ≤ 0.05 were considered statistically significant. The use of Wilcoxon-Mann-Whitney-U test was preferred to survival analysis in this study, because of the homogenous follow-up period for all included patients (see patient characteristics). Associations of outcomes with dichotomic variables were assessed in logistic regression models. After univariate analyses, multivariate analyses were performed for significant associations using a *P* value ≥ 0.05 for removal from the model. At least ten events per variable were considered reasonable in our analyses to avoid overfitting. Therefore, we only tested multiple combinations of two markers (with 28 events of acute decompensation recorded in this study). Receiver operating characteristic (ROC) analyses were performed to assess the capacity to predict AD from DAMP serum levels and “routine” biochemistry and hematology at baseline.

## Results

### Patient characteristics

In total, 160 patients with advanced-stage liver disease (cirrhosis) were included according to the above described inclusion criteria. Demographic and baseline characteristics of these patients are depicted in Table 1. Eighty patients (50%) were diagnosed with alcoholic liver cirrhosis (defined by a reported daily drinking average above 20g/dl in their patient history [34]), 60 patients (37.5%) with hepatitis C- and 20 patients (13.5%) with hepatitis B virus-associated liver cirrhosis. Sixty-nine patients with alcoholic liver cirrhosis (86.2%) reported no alcohol consumption for at least four consecutive years (as assessed by thorough anamnesis). One hundred fifteen patients showed stable compensated liver disease (cCLD) throughout this study (71.9%). The median follow-up time was 721 days. Seventeen patients were diagnosed with decompensated liver disease (dCLD) at baseline (10.6%) (see S5 Fig) from which 11 patients (57.9%) were diagnosed with alcoholic liver disease. Only two patients reported ongoing alcohol abuse (18.2%). From 28 patients with AD during follow-up, 19 (57.9%) were diagnosed with alcoholic liver disease with ongoing alcohol abuse in 5 out of 19 (26.3%). During follow-up, 28 patients (17.5%) developed acute decompensation (AD). Development of ascites was the most common cause of AD with 29 over 42 registered decompensations (64.4%). Hepatic encephalopathy and gastrointestinal hemorrhage were diagnosed in 8 patients (17.8%) und 5 patients (11.1%), respectively. Five patients (11.1%) developed acute-on-chronic liver failure (ACLF) according to the EF-CLIF consortium [35], with a mortality of 80% in this study. Overall, thirteen patients (8%) died during follow-up (Table 2) and five patients were lost to follow up before completing the two year follow up time frame.

**Table 1:** Baseline characteristics of included patients.

Characteristics	Cohort (N = 160)
Male gender, n (%)	94 (58.8)
Age (years), median (IQR)	62 (57–68)
BMI (kg/m^2^), median (IQR)	26,12 (23.34–29.32)
ARFI (m/sec), median (IQR)	2.72 (2.24–3.4)
Alcoholic cirrhosis, n (%)	80 (50.0)
HCV cirrhosis^#^, n (%)	60 (37.5)
HBV cirrhosis*, n (%)	20 (12.5)
Bilirubin (mg/dL), median (IQR)	1 (0.6–1.9)
ALT (U/l), median (IQR)	26.5 (19.25–36)
AST (U/l), median (IQR)	37.5 (29–51)
GGT (U/l), median (IQR)	57 (33–134)
Sodium (mmol/l), median (IQR)	140 (138–141)
Albumin (g/dL), median (IQR)	4.1 (3.5–4.5)
INR, median (IQR)	1.16 (1.07–1.33)
Platelets (/nl), median (IQR)	125 (87.5–177.75)
Child-Pugh A / B / C, n (%)	120 (75.0) / 37 (23.1) / 3 (1.9)
cCLD, n (%)	115 (71.9)
dCLD (at baseline), n (%)	17 (10.6)
AD, n (%)	28 (17.5)
Death, n (%)	13 (8.1)
Follow-up (days), median (min—max)	720.5 (242–898)

AD, acute decompensation; ALT, alanine aminotransferase; AST, aspartate aminotransferase; ARFI, acoustic radiation force imaging (m/sec); cCLD, compensated liver disease; dCLD, decompensated liver disease; GGT, gamma-glutamyl transferase; HBV, hepatitis B virus; HCV, hepatitis C virus, INR, international normalized ratio; IQR, interquartile range.

Most patients with viral hepatitis-related liver cirrhosis showed low or undetectable viral load

*HBV with 75% of patients on antiviral treatment and 20% showing detectable viral load, ≤ 50 IU/ml)

^#^HCV with documented sustained virological response in 86% (data not shown).

**Table 2:** Characteristics of patient subgroups according to compensated or decompensated liver disease.

Characteristics	cCLD (N = 115)	dCLD (baseline) (N = 17)	*P*	AD (N = 28)	*P*
Male gender, n (%)	68 (59.1)	11 (64.7)	-	15 (53.6)	-
Age (years), median (IQR)	61 (54–66)	62 (58–67)	n.s.	61 (56–67)	n.s.
BMI (kg/m^2^), median (IQR)	26.56 (24.07–29.39)	23.45 (21.67–29.19)	n.s.	24.25 (21.37–28.96)	0.049
Alcoholic cirrhosis, n (%)	51 (63.8)	10 (12.5)	-	19 (23.8)	-
HCV cirrhosis, n (%)	49 (81.7)	6 (10)	-	5 (8.3)	-
HBV cirrhosis, n (%)	15 (75)	1 (5)	-	4 (20)	-
Bilirubin (mg/dl), median (IQR)	0.8 (0.5–1.6)	1.6 (0.7–2.5)	0.02	1.7 (0.7–3.18)	<0.001
ALT (U/l), median (IQR)	27 (20–36)	23 (15.5–31)	n.s.	26.5 (17.75–36.75)	n.s.
AST (U/l), median (IQR)	34 (27–45)	48 (39–54.5)	<0.01	47.5 (33–73)	<0.01
GGT (U/l), median (IQR)	50 (30.75–91)	84 (43.5–137.5)	0.04	181.5 (46–342.5)	<0.01
Sodium (mmol/l), median (IQR)	140 (138–141)	136 (132.5–138.5)	<0.001	138 (135–142)	n.s.
Albumin (g/dl), median (IQR)	4.3 (3.8–4.5)	3.3 (3–3.75)	<0.001	3.7 (3.2–3.98)	<0.001
INR, median (IQR)	1.11 (1.05–1.24	1.28 (1.1–1.44)	<0.01	1.3 (1.13–1.44)	<0.01
Platelets (/nl), median (IQR)	129 (97–180)	154 (104.05–230)	n.s.	90 (76–140)	0.01
Death, n (%)	1 (7.7)	4 (30.8)	-	8 (61.5)	-

AD, acute decompensation; ALT, alanine aminotransferase; AST, aspartate aminotransferase; ARFI, acoustic radiation force imaging (m/sec); cCLD, compensated liver disease; dCLD, decompensated liver disease; GGT, gamma-glutamyl transferase; HBV, hepatitis B virus; HCV, hepatitis C virus, INR, international normalized ratio; IQR, interquartile range; n.s., not significant. p-values describe significance between cCLD vs. dCLD and cCDL vs. AD.

### DAMPs are elevated in decompensated liver disease

In the present study, serum levels of DAMPs were assessed in patients with chronic liver disease (compensated/cCLD *versus* decompensated liver disease/dCLD) at baseline (case-control study). First, we compared characteristics/etiology and routine biochemistry in cCLD patients with dCLD patients (Table 2). Alcoholic cirrhosis was more common than viral hepatitis-related liver cirrhosis in dCLD (64.4% and 35.6%, respectively). DAMP serum levels (i.e. IL-6, cyt C, sFasL, HMGB1 and CK18-M30 and -M65) were then analyzed between those two patient groups. Patients with dCLD at baseline showed significantly higher serum levels of IL-6 (*P*<0.001), cyt C (*P* <0.01), CK18-M65 (*P*<0.001) and–M30 (*P* <0.001) than patients with cCLD (Table 3). HMGB1 and sFasL were not statistically different between cCLD and dCLD (Table 3).

**Table 3:** Serum levels of DAMPs in compensated and decompensated liver disease.

DAMPs	cCLD	dCLD (baseline)	*P*	AD (during FU)	*P*
IL-6*	16.88 (9.8–42.7)	95.35 (54.2–225.5)	<0.001	40.27 (22.7–72.1)	<0.01
sFasL*	65.1 (45.3–88.9)	68.4 (42.3–110.6)	n.s.	88 (57.8–108.7)	<0.01
cyt C*	493 (200.4–1109.8)	1289.8 (534.7–1673.8)	<0.01	728.6 (362.9–1373.5)	0.047
HMGB1*	20390.3 (11177.6–34077.4)	18746.8 (8650.7–26421.8)	n.s.	31177.1 (18266–42707.3)	0.03
CK18-M30^#^	165.9 (112–288.1)	220.1 (189.3–326.6)	0.01	292.3 (196.3–379.5)	<0.01
CK18-M65^#^	381.9 (267.8–598.9)	595.8 (422.3–1240.7)	<0.01	623.5 (473.6–823)	<0.001

* (pg/ml), median (IQR)

^#^ (U/l), median (IQR); p-values describe significance between cCLD vs. dCLD and cCDL vs. AD.

No differences in serum marker concentration were found in viral hepatitis-associated liver cirrhosis between HBV and HCV (see S1 and S2 Figs). Noteworthy, several “routine” biochemistry data showed significant differences, i.e. gamma-glutamyl transferase (GGT; *P*<0.04), whereas alanine aminotransferase (ALT) levels were not significantly different between cCLD and dCLD (Table 2).

### DAMPs are predictors of acute decompensation in chronic liver disease

**Fig 1:**
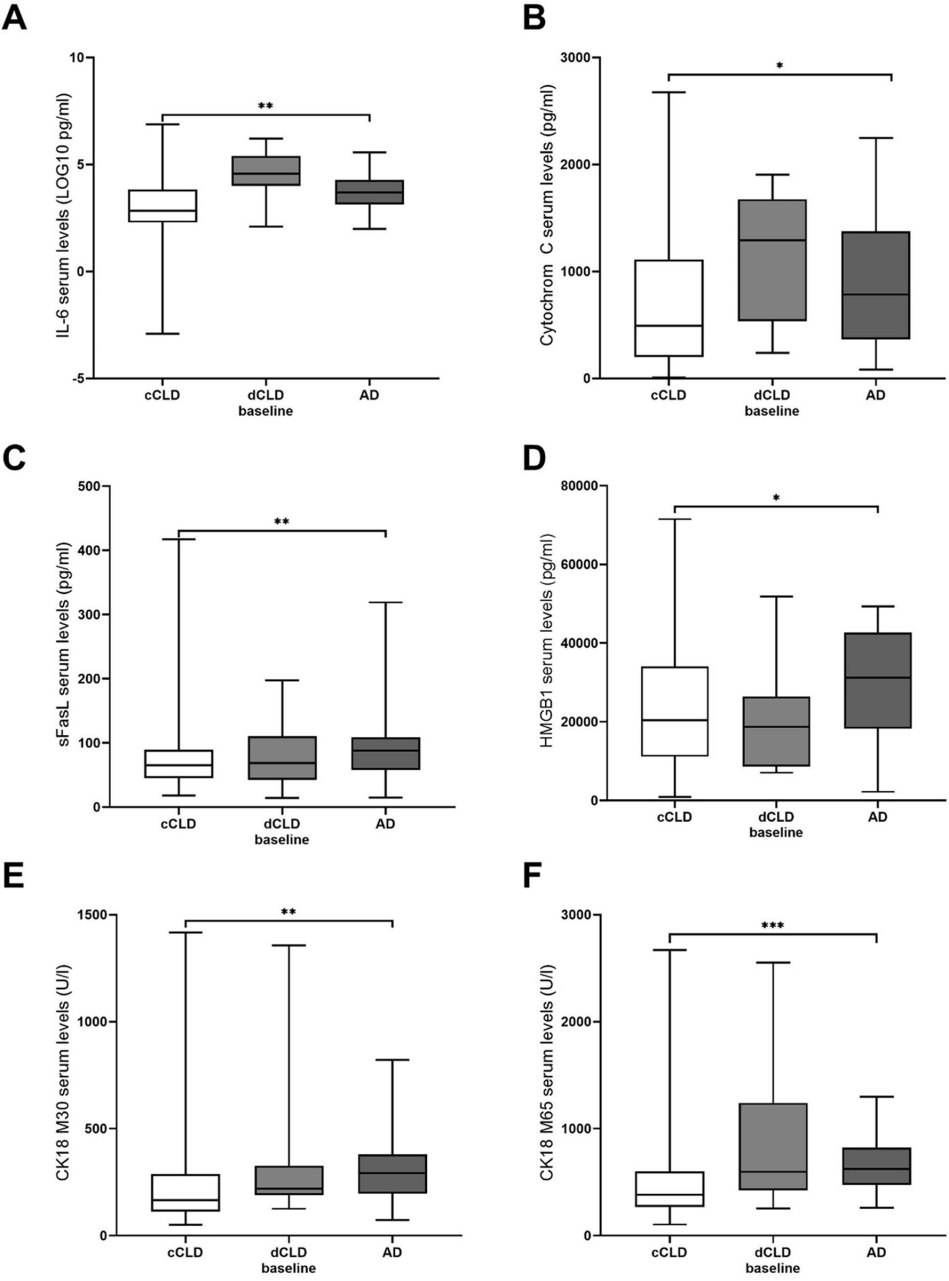
Comparison of baseline serum levels. (A) interleukin 6, (B) cytochrom C, (C) sFasL, (D) HMGB1, (E) CK18-M30 and–M65 and (F) ALT levels in CLD patients and patients with AD during follow-up. Box plots display the median and 25%- and 75%-quartiles. (* = p≤0.05; ** = p≤0.01; *** = p≤0.001).

The above described analyses suggest an association between elevated serum levels of DAMPs and dCLD. To further explore this relationship and to assess the potential predictive value of DAMPs for acute decompensation (AD), uni- and multivariate regression analyses were performed. We explored baseline DAMP levels and “routine” biochemistry data in 115 patients with stable compensated CLD (71.9%) and 28 patients with CLD and AD during follow-up (17.5%). Patients with dCLD at baseline were excluded from this analysis (n = 17; 10.6%). We find significantly elevated serum levels for most DAMPs in patients developing AD during follow up compared to patients with stable cCLD. In particular, serum levels of IL-6, sFasL and the soluble cytokeratin CK18-M65 and its caspase‐cleaved fragment CK18-M30 were significantly elevated in AD (*P*<0.01) (Fig 1, Table 3). Univariate analysis (*P*, beta [SD beta]) showed the cytokeratin-18 fragments M30 (*P*<0.01, 0.84 [0.32]) and M65 (*P*<0.01, 1.02 [0.37]), IL-6 (*P*<0.01, 0.4 [0.16]) as well as bilirubin (*P*<0.001, 0.94 [0.28]) and gamma-glutamyl transferase (GGT) (*P*<0.001, 0.74 [0.21]) from “routine” biochemistry as strongest predictors of AD in patients with CLD. Interestingly, the serum level of HMGB1 was not different between patients with cCLD and dCLD at baseline but was significantly elevated in patients with compensated CLD developing AD during follow *versus* patients with stably compensated CLD (*P* = 0.03). A similar correlation could also be observed for sFasL (*P*<0.01) (Fig 1, Table 3) and cyt C (*P* = 0.04, 0.47 [0.23]) (Table 4). In multivariate analysis (*P*, beta [SD beta]), sFasL (*P* = 0.02, 0.99 [0.41]) and GGT (*P*<0.001, 0.74 [0.21]) were both significant predictors of AD during the follow-up of patients with CLD. Logistic regression analysis of baseline DAMPs and “routine” biochemistry achieved comparable results for the prediction of AD (see Table 4). A logistic regression model with stepwise backward elimination identified baseline levels of sFasL and GGT to independently predict AD in the follow-up of patients with CLD (*P*<0.001). Interestingly, ALT level at baseline was not an independent predictor of AD and could be deleted from the logistic regression model without a statistically significant loss of fit.

**Table 4:** Logistic regression analyses of patient characteristics, routine biochemistry and DAMPs to predict acute decompensation.

	Univariate analysis			Multivariate analysis		
	*P*	beta (SD beta)	OR (95% CI)	*P*	beta (SD beta)	OR (95% CI)
***Patient characteristics***						
Male gender	n.s.	-0.33 (0.43)	0.72 (0.31–1.65)			
Age (years, cont.)	n.s.	0.01 (0.02)	1.01 (0.97–1.05)			
BMI (kg/m^2^, cont.)	n.s.	- 1.97 (1.3)	0.14 (0.01–0.8)			
***Routine biochemistry***						
Albumin (g/dl, cont.)	<0.001	-1.83 (0.44)	0.16 (0.07–0.38)			
Bilirubin (mg/dl, cont.)	<0.001	0.94 (0.28)	2.55 (1.48–4.4)			
AST (U/l, cont.)	<0.01	1.29 (0.43)	3.64 (1.56–8.51)			
ALT (U/l, cont.)	n.s.	0.15 (0.43)	1.16 (0.5–2.72)			
GGT (U/l, cont.)	<0.001	0.74 (0.21)	2.1 (1.38–3.18)	<0.001	0.74 (0.21)	2.1 (1.38–3.18)
Sodium (mmol/l, cont.)	0.03	-0.2 (0.1)	0.8 (0.67–0.98)			
INR (cont.)	n.s	0.27 (0.43)	1.31 (0.56–3.05)			
Creatinine (mg/dl, cont.)	n.s.	1.06 (0.7)	2.88 (0.72–11.52)			
Platelets (/nl, cont.)	n.s.	-0.48 (0.32)	0.62 (0.33–1.15)			
***DAMPs***						
IL-6 (pg/ml, cont.)	0.01	0.4 (0.16)	1.5 (1.09–2.06)			
cyt C (pg/ml, cont.)	0.04	0.47 (0.23)	1.6 (1.02–2.5)			
sFasL (pg/ml, cont.)	0.02	0.95 (0.41)	2.59 (1.16–5.78)	0.02	0.99 (0.41)	2.67 (1.19–6.05)
HMGB1 (pg/ml, cont.)	n.s.	0.47 (0.3)	1.6 (0.88–2.9)			
CK18-M30 (U/l, cont.)	<0.01	0.84 (0.32)	2.33 (1.24–4.35)			
CK18-M65 (U/l, cont.)	<0.01	1.02 (0.37)	2.77 (1.35–5.67)			

ALT, alanine aminotransferase; AST, aspartate aminotransferase; CK18-M30, cytokeratin 18 fragment M30; CK18-M30, cytokeratin 18 fragment M30; cyt C, cytochrome C; GGT, gamma-glutamyl transferase; HMGB1, high-mobility group-box protein 1; IL-6, interleukin 6; INR, international normalized ratio; sFasL, soluble Fas-ligand; cont., continuous. Logarithmic values were used for this analysis.

### Predictive capacity for acute decompensation

**Fig 2:**
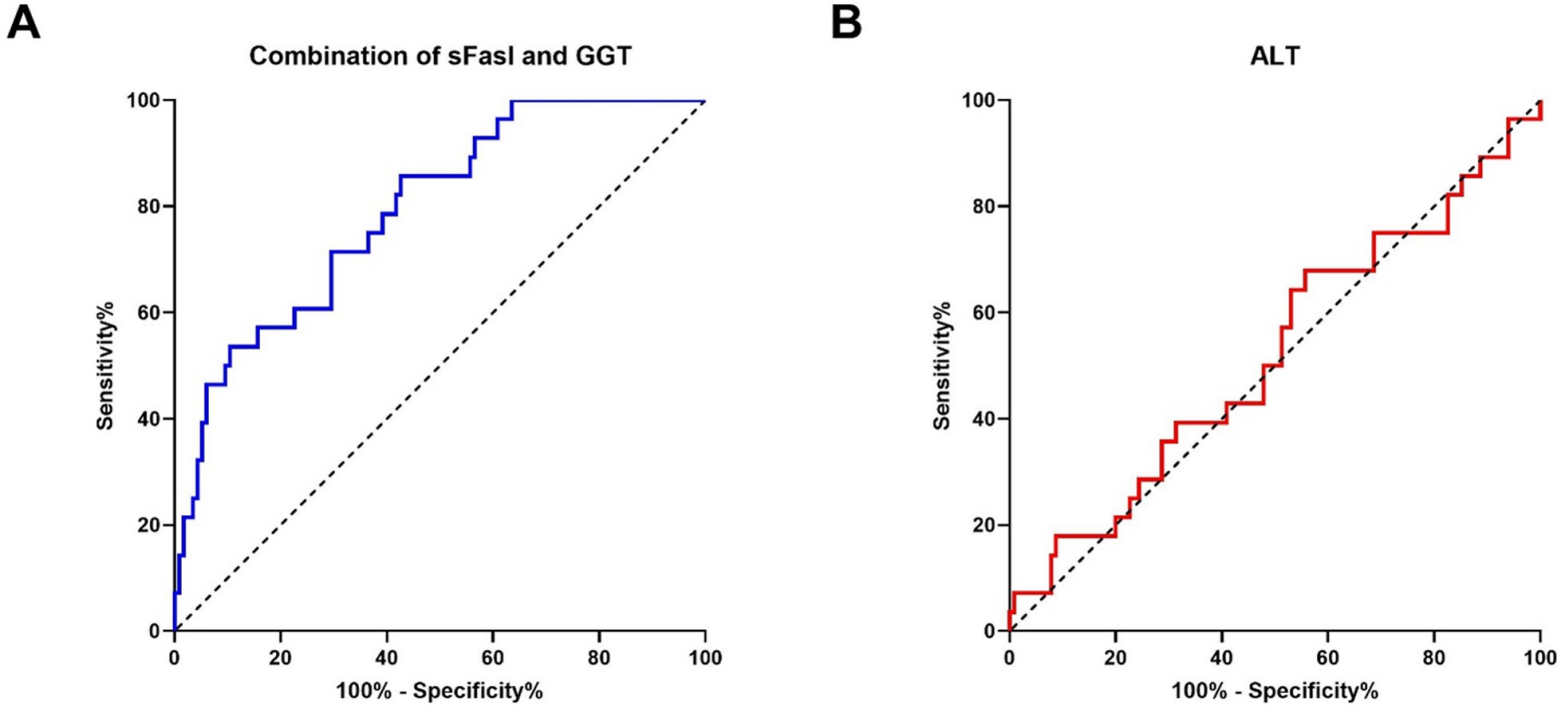
Area under the receiver operating characteristics (AUROC). (A) the marker combination sFasL plus GGT predicting the risk for AD during follow-up and (B) ROC curve for baseline ALT levels without predictive capacity for AD.

Receiver operating characteristic (ROC) analyses were performed to assess the capacity of baseline levels of DAMPs and “routine” biochemistry to predict AD in the surveillance of patients with CLD. In AUROC analyses (Area Under the Receiver Operating Characteristics), DAMPs showed at least AUC ≥ 0.62 (see S3 Fig). Based on the logistic regression model (see above), the combination of sFasL and GGT achieved an AUC of 0.79 with a specificity of 0.9 at optimal cut-off (see S4 Fig). ALT had no class separation capacity (AUC = 0.51) (Fig 2). For benchmarking, we calculated the AUC in predicting AD for the Model of End-Stage Liver Disease score MELD-Na, a scoring system associated with mortality in patients with advanced-stage liver disease and cirrhosis [33]. The AUC for MELD-Na to predict AD was 0.68, which was inferior to the marker combination sFasL and GGT, although the difference was not statistically significant in direct comparison of the respective AUCs.

## Discussion

Blood markers suitable for surveillance in patients with advanced-stage liver disease and predicting patients at risk for hepatic decompensation are urgently needed. To date, serum aminotransferase levels are widely used as surrogate markers for liver inflammation, however, ALT is difficult to interpret and frequently fails to identify patients with ongoing hepatic injury [11]. Serum ALT activity is previously reported as independently related to body mass index, hepatic steatosis and non-alcoholic steatohepatitis (NASH). Given the high prevalence of NASH in the western world, ALT elevation is often observed, however, its clinical importance is contentious [36, 37]. Moreover, there is an ongoing discussion about the normal range of aminotransferase levels in chronic liver disease. Prati and coworkers suggested a revision of the upper normal limits for ALT in patients with chronic HCV infection or non-alcoholic fatty liver disease (NAFLD) [38]. Recently we reported ongoing liver inflammation in about one third of patients with chronic HCV and sustained virological response upon antiviral treatment [39]. Twenty-five percent of those patients had normal ALT levels but showed aminotransferase activity above the so-called healthy range. Overall, the correlation of ALT elevation with ongoing inflammation is only weak, and cannot be considered a reliable predictor for disease progression.

However, systemic inflammation increases across distinct stages of chronic liver disease and is reported to correlate with decompensation and mortality [40, 41]. Hepatic cell death is accompanied by sterile inflammation that can cause ongoing liver damage and worsening of liver cirrhosis [42] and likely perpetuate a self-sustaining vicious cycle. Damage-associated molecular patterns released from dying cells, DAMPs, are considered a molecular link between cell death and inflammation [16, 43]. DAMPs interact with receptors of the innate immune system, similar to those targeted by bacterial compounds (pathogen-associated molecular patterns, PAMPs) and initiate complex intertwined mechanisms that lead to inflammatory reactions. In the present study, we characterized the level of DAMPs in sera from patients with stably compensated liver disease and patients with decompensated chronic liver disease to identify markers that correlate with decompensation. The marker panel tested in our study reflects apoptotic and necrotic cell death, both previously reported to play a role liver disease progression [16]. Noteworthy, although DAMPs have been associated with various liver diseases in previous studies, quantification of serum levels alone does not allow conclusions to be drawn about their site of origin (markers are not specific to hepatocytes but may also indicate an injury of other tissues) [44, 45].

The major finding from our study is that markers of cell death are elevated in decompensated *versus* compensated liver disease and potentially predict patients at risk for acute decompensation in the surveillance of chronic liver disease. Thereby, serum levels of DAMPs were significantly elevated irrespective of the underlying disease etiology. This suggests that although the pathophysiology and mechanism of liver diseases are different, necrotic and apoptotic cell death is upregulated in patients developing decompensated liver disease. Here, we characterized specific markers of cell death as predictors of disease progression and show their superiority over the currently used routine marker for necroinflammation, ALT.

We observed significantly elevated serum levels of DAMPs already in compensated liver disease as early as 2.5 years prior to hepatic decompensation. Thereby, several cell death markers were independently associated with acute decompensation, that is interleukin 6, cytochrome C and soluble Fas-ligand. In line with previous observations in patients with acute-on-chronic liver failure [31], we find an association of cytokeratin-18 and its caspase‐cleaved fragments with the development of acute decompensation in patients with chronic liver disease. As part of the cytoskeleton and characterized by its pervasive occurrence in cells, cytokeratin-18, in our study, was among the two strongest predictors for hepatic decompensation together with interleukin 6, a major pro-inflammatory cytokine released from macrophages. Pathophysiology, release kinetics and turnover of DAMPs might explain differences in serum levels and need further investigation to better interpret their role in liver disease progression. Noteworthy, we found no correlation between acute decompensation and levels of ALT in patients with liver cirrhosis. By using a logistic regression model, we identified that serum levels of soluble Fas-ligand and GGT independently predict the risk of decompensation during follow-up of patients with compensated chronic liver disease. Interestingly, levels of sFasL differed significantly between the cCLD and AD groups but not between the cCLD and dCLD groups. One possible explanation for these seemingly paradoxical results could be that sFasL is upregulated in chronic liver disease patients who are still compensated. Noteworthy, sFasL competitive binding to Fas receptor produces anti-apoptotic and anti-cell death effects in nuclei of target cells via activation of pro-survival signaling cascades. In the decompensated liver cirrhosis group, this cellular stress response might be attenuated or abolished due to the progressive loss of function of the cells [46–48]. Regarding our observations on the GGT, previous studies already found GGT levels paralleled with elevated serum ferritin levels [49] to correlate with liver inflammation in patients with cirrhosis [50]. Because ferritin was not part of the routine biochemistry in our patient cohort, we could not reassess this observation in our patients. Although the underlying mechanism is not known, GGT seems a better predictor of hepatic decompensation than ALT, which is also shown in HCV-infected patients upon antiviral treatment and sustained virological response [51].

Accuracy of serum Fas-ligand and GGT levels for predicting acute decompensation in the follow-up of patients with compensated liver disease was high (AUC = 0.79, *p* < 0.001). To compare the predictive capability of our marker combination with a clinical score that is routinely used in patients with end-stage liver disease, we calculated MELD-Na [33]. The score is based on serum bilirubin, creatinine, sodium and international normalized ratio for prothrombin time and considered a reliable measure of mortality risk in patients with end-stage liver disease. Importantly, we find that the accuracy of Fas-ligand and GGT serum levels in predicting acute decompensation outperformed the prediction accuracy of the MELD-Na score in our patients. Noteworthy, MELD-Na comprises parameters aiming at liver and kidney function rather than necroinflammation and cell death. However, our data suggest that cell death markers have the capability to improve currently used prognostic scores in the surveillance of patients with chronic liver disease.

Strengths of our study are the number of patients with advanced-stage liver disease included and the long observation period of 2.5 years. A limitation of the study is the small cohort size of patients developing acute decompensation during the observation period and the retrospective study design. Because of that, our data are “hypothesis-generating” warranting further prospective validation. The dynamics of cell death markers should be characterized in the clinical course of disease (comprising resolution of inflammation and recompensation) while monitoring various precipitants of acute decompensation (e.g. infections, alcohol abuse). Possibly, cell death markers could be useful tools to monitor the clinical course of disease and the response to therapy. Moreover, a “healthy” control group should be characterized in prospective studies to assess baseline levels of cell death markers. The etiology of the included patients in our study did not include NAFLD-induced cirrhosis. NAFLD is among the most important liver diseases with increasing prevalence in the Western world [52]. Because mechanisms of cell death and release of DAMPs in fatty liver disease appear to be similar to those in alcoholic liver disease [16], extrapolation of the results from patients with alcoholic liver disease from our study to NAFLD patients may be possible. However, this needs to be validated in follow-up studies.

In conclusion, this is the first study with a comprehensive panel of cell death markers investigating their capacity to predict patients at risk for the development of acute decompensation. Our data demonstrate a correlation between elevated serum levels of DAMPs and hepatic decompensation with potential implications for the management and surveillance of patients with chronic liver disease.

## Supporting information

S1 Fig Comparison of Interleukin-6 serum levels in HBV and HCV cirrhosis. (PDF)

S2 Fig Comparison of cytochrome c serum levels in HBV and HCV cirrhosis. (PDF)

S3 Fig Comparison of different DAMP AUCs. (PDF)

S4 Fig ROC analysis for mathematically combined marker. (PDF)

S5 Fig Flowchart of the study design. (PDF)

S6 Fig Comparison of DAMP serum levels in viral and alcoholic liver cirrhosis. (PDF)

S7 Fig Correlation between different cell death markers. (PDF)

S8 Fig AUCs of routine chemistry. (PDF)

## Abbreviations

ACLF: acute-on-chronic liver failure
AD: acute decompensation
ALT: alanine aminotransferase
AST: aspartate aminotransferase
AUC: area under the curve; BMI, body mass index; CK18, cytokeratin 18; CLD, chronic liver disease; cyt C, cytochrome C
DAMP: damage-associated molecular pattern
EDTA: ethylenediaminetetraacetic acid
ELISA: enzyme-linked immunosorbent assay
GGT: gamma-glutamyl transferase
HBV: hepatitis B virus
HCC: hepatocellular carcinoma
HCV: hepatitis c virus
HIV: human immunodeficiency virus
HMGB1: high-mobility group-box protein 1
IL-6: interleukin 6
INR: international normalized ratio for prothrombin time
IQR: interquartile range
MELD: model of end-stage liver disease
PAMP: pathogen-associated molecular pattern
sFasL: soluble Fas-ligand
SWE: shear-wave elastography
