# Supplementary material for: Diagnostic and prognostic significance of cell death markers in patients with cirrhosis and acute decompensation
Source: PLoS One. 2022 Feb 17;17(2):e0263989. doi: 10.1371/journal.pone.0263989 (PMC8853504; doi:10.1371/journal.pone.0263989)
Supplement: S1 Fig — (PDF) [file pone.0263989.s001.pdf]

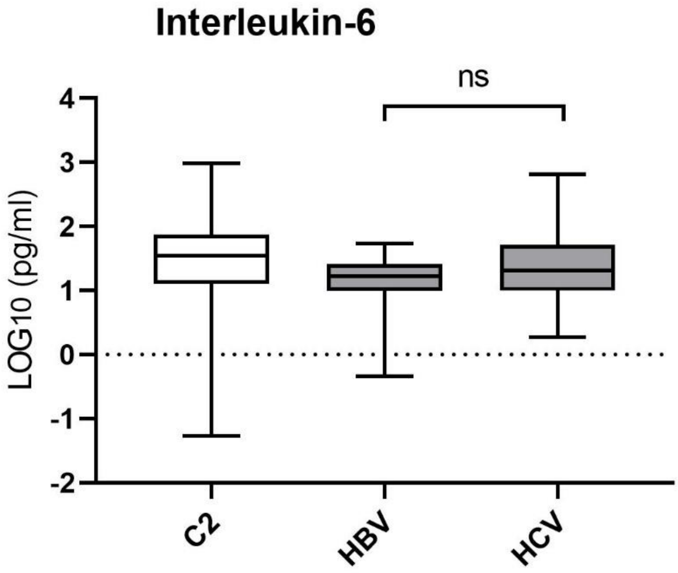

S1 Fig. Comparison of Interleukin-6 serum levels in HBV and HCV cirrhosis. No difference was found between HBV and HCV serum levels. (C2 = alcoholic liver cirrhosis, HBV = Hepatitis B associated liver cirrhosis, HCV = Hepatitis C associated liver cirrhosis, ns= not significant)
