# Supplementary material for: Diagnostic and prognostic significance of cell death markers in patients with cirrhosis and acute decompensation
Source: PLoS One. 2022 Feb 17;17(2):e0263989. doi: 10.1371/journal.pone.0263989 (PMC8853504; doi:10.1371/journal.pone.0263989)
Supplement: S3 Fig — (PDF) [file pone.0263989.s003.pdf]

Marker	Area under Curve (AUC)	Best cut-off	Sensitivity at Cut-off	Specificity at Cut-off
Cytochrom C	0.62	755.525	0.57	0.68
IL-6	0.68	18.165	0.89	0.52
sFasL	0.66	85.685	0.57	0.74
HMGB1	0.63	28271.35	0.61	0.68
ALT/GPT	0.51	n.a.	n.a.	n.a.

S3 Fig. Comparison of different DAMP AUCs
