# Supplementary material for: Diagnostic and prognostic significance of cell death markers in patients with cirrhosis and acute decompensation
Source: PLoS One. 2022 Feb 17;17(2):e0263989. doi: 10.1371/journal.pone.0263989 (PMC8853504; doi:10.1371/journal.pone.0263989)
Supplement: S4 Fig — (PDF) [file pone.0263989.s004.pdf]

Marker combination	Area under Curve (AUC)	Best cut-off	Sensitivity at Cut-off	Specificity at Cut-off
GGT and sFasL	0.79	-24.78	0.54	0.9
Formula for marker combination resulted from logistic regression:<br>-12,672 + (-1,0285*GGT serum levels) + (-1,556*sFasL serum levels)<br>(Of note: the resulting score is unitless therefore allowing a negative cut-off)				

S4 Fig. ROC analysis for mathematically combined marker
