# Supplementary figures and images for: Diagnostic and prognostic significance of cell death markers in patients with cirrhosis and acute decompensation
Source: PLoS One. 2022 Feb 17;17(2):e0263989. doi: 10.1371/journal.pone.0263989 (PMC8853504; doi:10.1371/journal.pone.0263989)

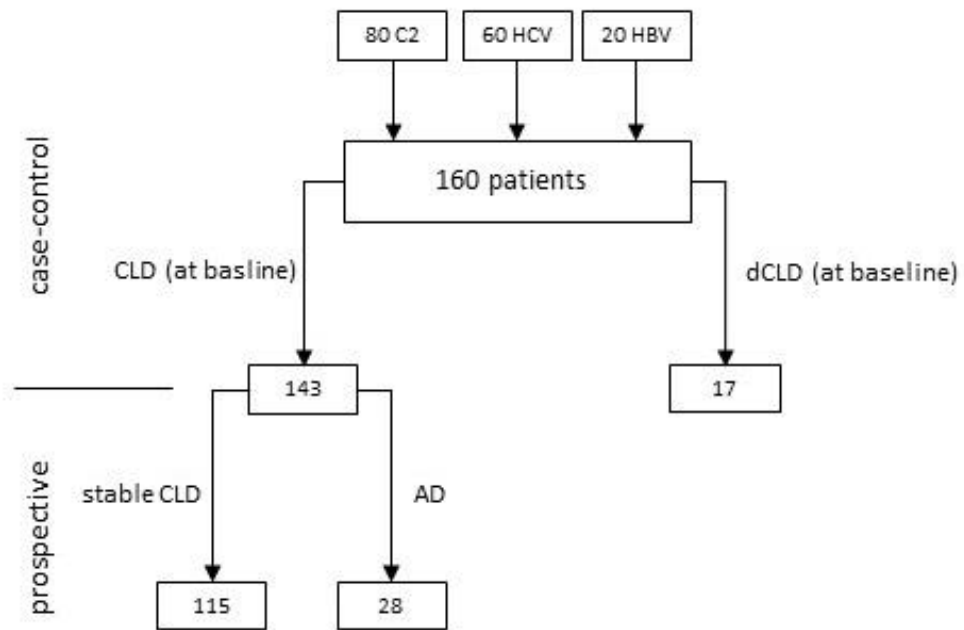

**S5 Fig. Flowchart of the study design**

Supplement: S5 Fig — (PDF) [file pone.0263989.s005.pdf]
