# Supplementary material for: Diagnostic and prognostic significance of cell death markers in patients with cirrhosis and acute decompensation
Source: PLoS One. 2022 Feb 17;17(2):e0263989. doi: 10.1371/journal.pone.0263989 (PMC8853504; doi:10.1371/journal.pone.0263989)
Supplement: S6 Fig — (PDF) [file pone.0263989.s006.pdf]

| Marker | Alcoholic cirrhosis | Viral cirrhosis | p-value |
|---|---|---|---|
| Cytochrom C* | 480.95 (252.44 – 966.07) | 740.64 (301.29 – 1449.06) | 0.02 |
| IL-6* | 35.4 (12.83 – 75.48) | 18.62 (9.84 – 48.96) | 0.01 |
| sFasL* | 65.5 (44.73 – 98.62) | 69.06 (50.97 – 93) | n.s. |
| HMGB1* | 19454.15 (10953.26 – 35283.5) | 23888.6 (12234.41 – 35223.04) | n.s. |
| CK-18 M30* | 217.32 (149.13 – 340.17) | 149.53 (94.57 – 293.94) | < 0.01 |
| CK-18 M65* | 496.69 (340.13 – 775.91) | 425.96 (252.56 – 607.18) | 0.01 |
| GPT# | 26 (18 – 36) | 27 (20 – 36) | n.s. |
| GGT# | 78 (48 – 225) | 40.5 (26 – 81.75) | < 0.01 |

**S6 Fig. Comparison of DAMP serum levels in viral and alcoholic liver cirrhosis**. (viral = HBV and HCV liver cirrhosis; * (pg/ml), median (IQR) # (U/l), median (IQR))
