# Supplementary material for: Diagnostic and prognostic significance of cell death markers in patients with cirrhosis and acute decompensation
Source: PLoS One. 2022 Feb 17;17(2):e0263989. doi: 10.1371/journal.pone.0263989 (PMC8853504; doi:10.1371/journal.pone.0263989)
Supplement: S7 Fig — (PDF) [file pone.0263989.s007.pdf]

	GGT	HMGB1	IL-6	Cytc	sFasL	M30	M65
GGT	1.0000	-0.0839	0.1128	0.0628	-0.1002	0.4810	0.5074
	.	0.3258	0.1813	0.4576	0.2352	0.0000*	0.0000*
HMGB1	-0.0839	1.0000	0.0226	0.2655	0.2864	0.1221	0.1016
	0.3258	.	0.7904	0.0016*	0.0006*	0.1504	0.2319
IL-6	0.1128	0.0226	1.0000	0.0480	0.2797	0.2446	0.2377
	0.1813	0.7904	.	0.5689	0.0007*	0.0033*	0.0043*
Cytc	0.0628	0.2655	0.0480	1.0000	0.1884	0.1920	0.2864
	0.4576	0.0016*	0.5689	.	0.0244*	0.0217*	0.0006*
sFasL	-0.1002	0.2864	0.2797	0.1884	1.0000	0.1208	0.1694
	0.2352	0.0006*	0.0007*	0.0244*	.	0.1505	0.0433*
M30	0.4810	0.1221	0.2446	0.1920	0.1208	1.0000	0.8029
	0.0000*	0.1504	0.0033*	0.0217*	0.1505	.	0.0000*
M65	0.5074	0.1016	0.2377	0.2864	0.1694	0.8029	1.0000
	0.0000*	0.2319	0.0043*	0.0006*	0.0433*	0.0000*	.

S7 Fig. Correlation between different cell death markers. (IL-6 = Interleukin 6, Cytc = Cytochrome C, M30 = CK18-M30, M65 = CK18-M65). The upper values are the correlation coefficients (rho) with the corresponding p-values in the lower row (e.g. correlation between GGT and HMGB1 rho: -0.0839, p=0.3258)
