# Supplementary material for: Diagnostic and prognostic significance of cell death markers in patients with cirrhosis and acute decompensation
Source: PLoS One. 2022 Feb 17;17(2):e0263989. doi: 10.1371/journal.pone.0263989 (PMC8853504; doi:10.1371/journal.pone.0263989)
Supplement: S8 Fig — (PDF) [file pone.0263989.s008.pdf]

Marker	Area under Curve (AUC)	Best cut-off	Sensitivity at Cut-off	Specificity at Cut-off
Albumin	0.78	4.25	0.93	0.56
Bilirubin	0.71	0.75	0.86	0.47
INR	0.68	1.28	0.54	0.78
ALT/GPT	0.51	24.5	0.64	0.44
AST/GOT	0.69	43.5	0.5	0.78
GGT	0.7	154	0.57	0.86

S8 Fig. AUCs of routine chemistry
